# Evaluation of Patients with Vaccine Allergies Prior to mRNA-Based COVID-19 Vaccination

**DOI:** 10.3390/vaccines10071025

**Published:** 2022-06-27

**Authors:** Xin Rong Lim, Justina Wei Lynn Tan, Grace Yin Lai Chan, Jinfeng Hou, Linlin Xie, Vivian Hui Li Goh, Joewee Boon, Samuel Shang Ming Lee, Claire Min-Li Teo, Sze Chin Tan, Khai Pang Leong, Bernard Yu Hor Thong, Bernard Pui Lam Leung

**Affiliations:** 1 Department of Rheumatology, Allergy and Immunology, Tan Tock Seng Hospital, Singapore 308433, Singapore; justina_tan@ttsh.com.sg (J.W.L.T.); grace_yl_chan@ttsh.com.sg (G.Y.L.C.); jinfeng_hou@ttsh.com.sg (J.H.); lin_lin_xie@ttsh.com.sg (L.X.); vivian_hl_goh@ttsh.com.sg (V.H.L.G.); joewee_boon@ttsh.com.sg (J.B.); samuel_lee@ttsh.com.sg (S.S.M.L.); claire_ml_teo@ttsh.com.sg (C.M.-L.T.); sze_chin_tan@ttsh.com.sg (S.C.T.); khai_pang_leong@ttsh.com.sg (K.P.L.); bernard_thong@ttsh.com.sg (B.Y.H.T.); bernard.leung@singaporetech.edu.sg (B.P.L.L.); 2 Health and Social Sciences, Singapore Institute of Technology, Singapore 138683, Singapore

**Keywords:** COVID-19 vaccine, vaccine allergies, polyethylene glycol, polysorbate

## Abstract

During the initial rollout of coronavirus disease 2019 (COVID-19) vaccination in Singapore, the Ministry of Health (MOH) issued a recommendation that patients with a history of any previous vaccine allergy be referred to an allergist for further review of their suitability to proceed with mRNA-based COVID-19 vaccines. Patients fulfilling the above criterion were divided into three groups: immediate reaction (Group A), delayed reaction (Group B) and no/irrelevant reaction (Group C). They were subjected to either a skin prick test (SPT) and intradermal test (IDT) with polyethylene glycol (PEG) or polysorbate-containing products; direct injection with the Pfizer BNT162b2 vaccine in the allergy clinic; or injection at community vaccination centres, respectively. Groups A and B were also invited to complete a questionnaire survey on post-vaccination reactions, and blood sampling pre-vaccination and 1 h after the first dose of the BNT162b2 vaccine to measure immunoglobulin (Ig) G, IgM and IgE antibodies to the Pfizer BNT162b2 vaccine via ELISA assays immobilised with the BNT162b2 vaccine, as well as levels of allergic cytokines interleukin (IL)-4 and IL-33, complement C5a and the endothelial activation marker intercellular adhesion molecule-1 (ICAM-1). Groups A and B comprised 62 (20.5%) patients each. In Group A, two subjects (3.2%) with equivocal IDT results tolerated both doses of the BNT162b2 vaccine without major allergic reactions. The remaining 60 (96.8%) in Group A and 62 (100%) in Group B completed both doses of BNT162b2 vaccination without major adverse reactions. Among the 99 who completed the questionnaire survey, 13 (13%) patients reported mild allergic reactions after the first dose of the vaccine. Immunoglobulin (Ig) G and M antibodies, but not IgE antibodies to the Pfizer BNT162b2 vaccine were detected in 67 subjects prior to vaccination. The presence of anti-Pfizer BNT162b2 IgG and IgM prior to vaccination did not result in major allergic reactions nor increases in Th2-related cytokines (IL-4, IL-33), complement activation products (C5a) or endothelial activation (ICAM-1). The majority of those with suspected reactions to non-COVID-19 polysorbate-containing vaccines tolerated the BNT162b2 vaccine. Excipient skin tests for PEG and polysorbate prior to vaccination are unnecessary.

## 1. Introduction

The coronavirus disease 2019 (COVID-19) pandemic has resulted in the unprecedented large-scale use of novel messenger ribonucleic acid (mRNA) vaccines packaged in lipid nanoparticles. Unlike conventional vaccines used, a small but significant number of severe allergic reactions have occurred with mRNA vaccines, sparking concern among both the population at large and healthcare authorities [1,2].

Phase 1 to 3 clinical trials of the Pfizer-BioNTech and Moderna mRNA vaccines have reported a low incidence of serious adverse events and equal incidence of adverse reactions in both vaccine and placebo groups [3,4]. However, these clinical trials excluded participants with a history of an allergic reaction to any component of the vaccine [4]. Since the rollout of the vaccinations worldwide, reports of severe hypersensitivity reactions post mRNA vaccinations surfaced, prompting various clinical immunology/allergy expert panels and professional bodies to issue guidance or recommendations that those with a history of an immediate allergic reaction to other vaccines or a previous dose of an mRNA COVID-19 vaccine or with a known allergy to any component of the mRNA vaccine be referred to an allergist-immunologist [5].

Singapore’s Health Sciences Authority (HSA) granted interim authorisation for the BNT162b2 vaccine under the Pandemic Special Access Route (PSAR) on 14 December 2020 [6] and for the Moderna vaccine on 3 February 2021 [7]. On 15 March 2021, Singapore’s Ministry of Health (MOH) issued a recommendation that patients with previous allergic reactions to non-COVID-19 vaccines be referred to an allergist for further review of their suitability to proceed with mRNA-based COVID-19 vaccines. To encourage vaccination uptake and mitigate the potential risks of adverse reactions to vaccines, these evaluations by allergists in public institutions were funded by the government. We review the evaluation of these patients with suspected vaccine allergies prior to receiving mRNA-based COVID-19 vaccines.

## 2. Materials and Methods

This cohort included 303 patients who were referred to the Tan Tock Seng Hospital Vaccine Allergy Clinic, Singapore, for evaluation of non-COVID-19 vaccine allergy prior to mRNA COVID-19 vaccination between 8 April 2021 and 22 September 2021.

The assessment and evaluation of the previous vaccine allergy included taking a comprehensive history of the details of the reaction to previous vaccination, reviewing past notes (if any), photo documentation (if available), laboratory results (if any), treatment given, and outcome as well as recovery. Reaction symptoms were classified as follows: immediate ≤4 h; delayed >4 h. Anaphylaxis was defined using the Brighton Collaboration Anaphylaxis Working Group’s case definition [8]. Relevant medical history, atopy history and drug history were also obtained during the evaluation.

Group A comprised patients with immediate hypersensitivity reactions to non-COVID polysorbate-containing vaccines; they proceeded to have a skin prick test (SPT) and intradermal test (IDT) with polyethylene glycol (PEG)-3350, polysorbate-80 and polysorbate-20-containing products (SPT/IDT group). At our centre, SPT and IDT were performed on the volar face of the arm using medroxyprogesterone acetate 50 mg/mL (containing polyethylene glycol (PEG-3350)/polysorbate-80), hepatitis A vaccine (Havrix) (containing polysorbate-20) and Optive Advanced UD eye drops (containing polysorbate-80) together with adequate positive (histamine) and negative (saline) controls according to methods from previously published reports [9]. Skin testing for the mRNA vaccine was not performed due to the scarcity of COVID-19 vaccines. Antihistamine was withheld for at least 72 h prior to the test. Wheal and erythema were measured at 15 min. A wheal size of greater than 3 mm was considered positive. Patients with a negative skin test were subjected to a single dose of the Pfizer vaccine, performed on a different day. Those with equivocal test results had a 2-step graded dose of the Pfizer vaccine performed 1 h apart after shared decision-making with the patients.

Group B included patients with delayed hypersensitivity reactions to polysorbate-containing vaccines, who proceeded to have a direct injection (DI) in the Allergy Clinic with the Pfizer BNT162b2 vaccine under the supervision of an allergist (DI group).

Group C comprised the remainder of patients with:

(1) non-allergic reaction to non-polysorbate-containing vaccines (e.g., expected side effects, subjective symptoms or local injection site reactions)
(2) previously tolerated polysorbate-containing vaccines, or
(3) no allergies to non-COVID vaccines.

They were reassured and advised to proceed with COVID-19 vaccination at community vaccination centres.

Groups A and B were also invited to complete a questionnaire survey on post-vaccination reactions, and blood sampling pre-vaccination and 1 h after the first dose of BNT162b2 vaccination to measure immunoglobulin (Ig) G, IgM and IgE antibodies to the Pfizer BNT162b2 vaccine and levels of allergic cytokines interleukin (IL)-4 and IL-33, complement C5a and endothelial activation marker intercellular adhesion molecule-1 (ICAM-1).

Those who were assessed as having an allergic reaction to non-COVID-19 vaccines were also allowed to proceed with COVID-19 vaccination at the community vaccination sites if the non-COVID-19 vaccine they were allergic to does not contain polysorbate or if the patient subsequently tolerated another polysorbate-containing vaccine.

**Figure 1:**
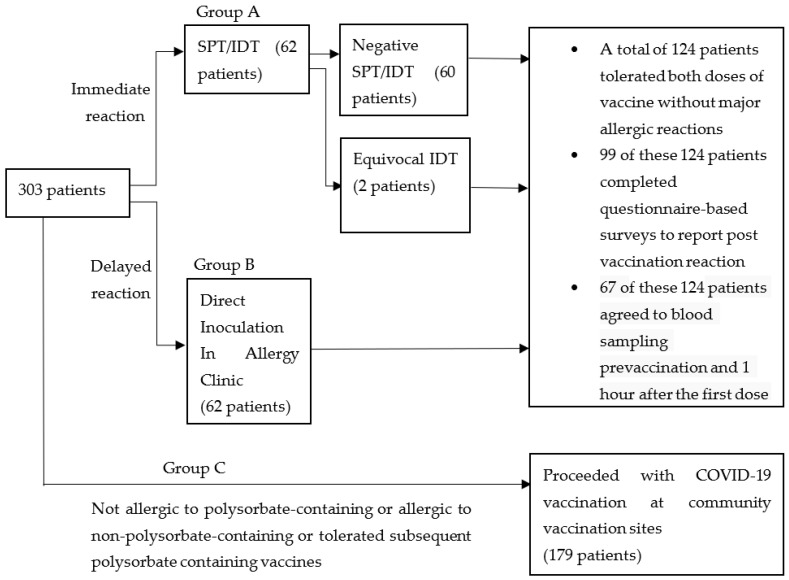
Grouping and evaluation strategy for patients referred for evaluation of non-COVID-19 vaccine allergies prior to mRNA COVID-19 vaccination.

The above is summarised in Figure 1.

Data collected were gathered from electronic health records reviewed by the authors. The structured clinical notes template used during the clinical consultations facilitated the data extraction. The data collected include patient demographics, details of the non-COVID-19 vaccine allergy, skin test results and outcome (whether the patient was able to tolerate 2 doses of the Pfizer BNT 162b2 vaccine without any allergic reactions).

Anti-Pfizer BNT162b2 IgE antibody titres of individual sera were detected with biotin-conjugated anti-human IgE (BD Biosciences, San Diego, USA) as previously described [10]; human anti-PEG IgE was employed as standard (0.45–1000 ng/mL) based on the supplier’s recommendation (Hu 6.3 IgE, Academia Sinica, Taipei, Taiwan). Anti-Pfizer BNT162b2 IgG (0.45–1000 ng/mL, Hu 6.3 IgG, Academia Sinica) and anti-Pfizer BNT162b2 IgM were measured in a similar manner [10]. Human complement 5a (C5a), interleukin (IL)-33, intercellular adhesion molecule-1 (ICAM-1) (R&D Systems, Abingdon, UK) and IL-4 (BD Biosciences) were assayed by enzyme-linked immunosorbent assay (ELISA) according to the manufacturers’ instructions.

The study was approved by the institutional review board (National Healthcare Group, Domain Specific Review Board reference number: 2021/00174), and written informed consent was obtained from participants in the SPT/IDT and DI groups who answered the questionnaire and agreed to blood sampling. A subsequent approval for waiver of informed consent for anonymised data collection from medical records of patients who did not answer the questionnaire was also given by our institution’s ethics board (DSRB 2021/00878).

### Statistical Analysis

Patient characteristics were summarised using descriptive analyses. Chi-square or Fisher exact tests were used for categorical variables, and Mann–Whitney U test for non-parametric variables. Data analyses were conducted using Prism 8 (GraphPad Software, San Diego, CA, USA), with *p* < 0.05 considered statistically significant.

## 3. Results

Of the 303 patients referred for evaluation, Group A comprised 62 (20.5%) patients with suspected immediate hypersensitivity reactions to non-COVID polysorbate-containing vaccines. Group B comprised 62 (20.5%) patients who reported delayed hypersensitivity reactions to polysorbate-containing vaccines and underwent direct inoculation in the Allergy Clinic. Group C comprised the remaining 179 (59.1%) individuals who were finally assessed as having a non-allergic reaction to non-polysorbate-containing vaccines, had previously tolerated polysorbate-containing vaccines or had no allergies to non-COVID vaccines (Figure 1).

There were 99 patients in Group A (SPT/IDT group) and Group B (DI group) who agreed to complete a questionnaire-based survey to report any post-vaccination reactions. A further 67 patients agreed to blood sampling pre-vaccination and 1 h after the first dose of the BNT162b2 vaccination.

### Group A (SPT/IDT) and Group B (DI) Cohort

Demographic characteristics, atopic diseases and reaction details of patients with polysorbate-containing vaccine allergies can be found in Table 1. Of the 124 patients, 82 (66.1%) were female and 88 (71%) were Chinese, with a mean age of 52.4 (SD ± 16.9) years. Forty-nine patients (40%) reported a concurrent drug allergy in addition to vaccine allergies. The most common atopic conditions were allergic rhinitis and asthma. In order of reported frequency, the type of reactions experienced after polysorbate-containing vaccinations were cutaneous (54.8%), lower airway (7.3%), cardiovascular (4.8%), upper airway (2.4%) and gastrointestinal (2.4%). One patient had suspected anaphylaxis to an influenza vaccine. She was placed in the DI cohort and received direct inoculation of the BNT162b2 vaccine without prior skin testing as she was unable to stop antihistamine therapy.

The types of vaccine that patients reported being allergic to are detailed in Table 2. Reported allergies to influenza and tetanus vaccines are the most common, with 54 (43.5%) patients allergic to influenza vaccines and 31 (25.8%) patients allergic to tetanus vaccines. Seventeen (13.7%) patients reported allergies to more than one vaccine. Four patients had reactions to non-polysorbate-containing vaccines but preferred to have skin testing or direct inoculation of the BNT162b2 vaccine at our centre.

All 62 patients who underwent SPT/IDT had negative results, except for 2 with equivocal IDT results. The first patient had a 2 mm wheal increment with surrounding erythema of 13 mm by 15 mm to medroxyprogesterone acetate 50 mg/mL at 1:10 concentration. The other had a 2 mm wheal increment with surrounding erythema of 11 mm by 19 mm to Optive Advanced UD eye drops at 1:10 concentration. Both patients tolerated a graded dose challenge of the Pfizer BNT vaccine, given as 0.1 mL followed by 0.2 mL 1 h later.

All 124 patients in the SPT/IDT and DI groups completed both doses of BNT162b2 vaccination without major adverse reactions. Of the 124 patients, 99 (79.8%) completed the questionnaire survey to report any post-vaccination reactions. Of the 99, 13 (13%) who completed the questionnaire reported reactions including non-specific rashes and mild urticaria/angioedema post the first dose of the vaccine. The reported reactions occurred more than 4 h after vaccine inoculation, with the exception of one within an hour of vaccine inoculation. All 13 patients subsequently completed the second dose of the BNT162b2 vaccine following consultation with an allergist, with 8 (61.5%) reporting similar mild skin reactions.

Similar to our previous findings, no detectable anti-Pfizer BNT 162b2 IgE was observed in any of the patients prior to vaccination [11]. IgG and IgM antibodies against the Pfizer BNT162b2 vaccine were detected in all patients, with mean (± SD) values of 372.66 ± 318.55 (ng/mL) and 25.19 ± 20.63 (arbitrary unit (AU)/mL), respectively. There was no significant elevation of IL-4, IL-33, ICAM-1 or C5a at 1 h post vaccination, and these levels did not differ in those who reported mild reactions compared with those who did not (Table 3).

## 4. Discussion

The messenger RNA (mRNA)-based COVID-19 vaccines (Pfizer-BioNTech and Moderna) use lipid nanoparticles to facilitate the transport of mRNA into cells. The novel excipient polyethylene glycol (PEG-2000) used to stabilise the lipid nanoparticle is recognised to have allergenic potential via IgE-mediated mechanisms or non-IgE mechanisms due to anti-PEG IgM or anti-PEG IgG antibodies or direct mast cell activation and complement activation of allergic effector cells [11,12]. However, to date, the exact cause of allergic reactions associated with mRNA COVID-19 vaccines remains unclear [13].

PEG and polysorbate are structurally similar, and hence cross-reactivity has been suggested. There was a concern that those who are allergic to polysorbate-containing vaccinations may develop reactions after receiving mRNA COVID-19 vaccines due to cross-reactivity between PEG and polysorbate. At the start of the COVID-19 vaccination drive, Banerji et al. recommended PEG and polysorbate skin testing for individuals with a reported allergic reaction to previous vaccines containing PEG or polysorbate or with a PEG/polysorbate allergy [9]. The authors advised that vaccination be withheld if PEG/polysorbate skin testing is positive, but that vaccination can proceed in those whose skin test is negative. To ensure safety and improve uptake of mRNA COVID-19 vaccinations, we carried out skin testing with PEG and polysorbate-containing products at concentrations previously reported to be non-irritating to exclude PEG and polysorbate allergies prior to mRNA-based COVID-19 vaccination.

Based on our skin testing results, despite having suspected allergies to polysorbate-containing non-COVID vaccines, all of the patients who underwent skin testing had negative SPT results, and only two patients had equivocal intradermal test results. All patients subsequently tolerated the Pfizer BNT162b2 vaccine without major allergic reactions. Most notably, the only patient who presented with anaphylactic symptoms after receiving the influenza vaccine had negative results for DPT, and she was able to tolerate both doses of the mRNA COVID-19 vaccine. This suggests that polysorbate allergies are rare and that excipient skin testing for PEG and polysorbate prior to receiving the mRNA vaccine is not cost-effective and not useful.

Other studies also provide additional data showing that excipient skin testing with PEG and polysorbate is of little utility in the assessment of suspected allergic reactions to SARS-CoV-2 vaccines [14]. Pitlick et al. demonstrated that despite having adverse reactions after the first dose of an mRNA vaccine and having a history of PEG allergies, the majority of patients had negative skin testing results, and 89.1% of them were able to tolerate subsequent vaccinations with no allergic symptoms, including 83% of those who had positive skin test results [15,16]. Similarly, Wolfson et al. reported that most individuals are able to receive the second dose of an mRNA COVID-19 vaccine after a reported allergic reaction to the first dose, regardless of skin test results [17]. Likewise, we demonstrate that patients can safely receive a second dose of the BNT162b2 vaccine despite reporting mild allergic reactions after the first dose.

Initial recommendations from Singapore’s Ministry of Health (MOH) on 15 March 2021 suggested that patients with allergic reactions to non-COVID-19 vaccines be referred to an allergist for further review of their suitability to proceed with mRNA-based COVID-19 vaccines. The intention then was to mitigate the risk of adverse reactions to a hitherto novel mRNA vaccine platform. An increased incidence of anaphylaxis induced by mRNA vaccines also could have potentially derailed the National Vaccination Program. However, Singapore’s MOH changed its recommendations subsequently on 27 October 2021 based on local and international data on the lack of utility of excipient skin testing prior to mRNA vaccination, advising that those who were allergic to non-COVID-19 vaccinations no longer required a pre-mRNA vaccination review by a specialist.

The prevalence of anti-PEG antibodies in healthy populations varies widely. Studies have shown that the prevalence ranges from 20% to 72% [18]. The presence of anti-PEG antibodies found in individuals who have had no exposure to PEGylated drugs suggests sensitization through other sources, presumably PEG and PEG derivatives in processed foods and personal care, beauty and household cleaning products [19]. Our novel ELISA assay to detect antibodies to the Pfizer BNT162b2 vaccine involves immobilising the Pfizer BNT162b2 vaccine on ELISA plates to allow the binding of antibodies in serum samples against all potential immunogenic epitopes of the vaccine. Emerging evidence has shown that PEG conjugated with lipid nanoparticles rather than PEG alone induces positive basophil activation in patients with a PEG allergy, suggesting that the structure or form of PEG plays a role in potentially triggering allergic reactions [20]. We demonstrate that anti-Pfizer BNT162b2 IgG and IgM were present prior to vaccination, yet these individuals did not exhibit major allergic reactions nor increases in Th2-related cytokines (IL-4, IL-33), complement activation products (C5a) or endothelial activation (ICAM-1). It still remains unclear whether anti-PEG IgM and IgG result in non-IgE-mediated reactions or complement activation-related pseudoallergy (CAPRA) in patients with mRNA vaccine allergies. It appears that the presence of these antibodies prior to mRNA COVID-19 vaccination does not induce reactions and that there could be other potential mechanisms, and genetic or epigenetic factors in place.

The weaknesses of our study include the potential recall bias among the patients with previous non-COVID-19 vaccine allergies. Skin testing was performed only with PEG and polysorbate-containing products and not with the Pfizer BNT162b2 vaccine due to the scarcity of COVID-19 vaccines.

## 5. Conclusions

The utility of excipient skin testing with polysorbate and PEG in the evaluation of polysorbate-containing vaccine allergies prior to mRNA COVID-19 vaccination is poor. Those who report a mild allergic reaction after the first dose of the Pfizer BNT162b2 vaccine can safely receive the second dose.

## Figures and Tables

**Table 1:** Demographic characteristics and reaction details of patients with non-COVID-19 polysorbate-containing vaccine allergies.

Demographics	SPT/IDT and DI Cohort *n* = 124	Suspected Immediate Hypersensitivity to Polysorbate-Containing Vaccines; SPT/IDT Cohort *n* = 62	Suspected Delayed Hypersensitivity to Polysorbate-Containing Vaccines; DI Cohort *n* = 62	*p*-Values
Age, mean (SD)	52.4 (16.9)	49.8 (16.0)	54.9 (17.6)	*p* = 0.091
Sex (female, %)	82 (66.1%)	44 (71%)	38 (61.3%)	*p* = 0.255
**Race (%)**				
Chinese	88 (71%)	44 (71%)	44 (71%)	*p* = 1.000
Malay	9 (7.3%)	5 (8%)	4 (6.5%)	*p* = 0.729
Indian	14 (11.3%)	6 (9.7%)	8 (12.9%)	*p* = 0.570
Others	13 (10.5%)	7 (11.3%)	6 (9.7%)	*p* = 0.769
**Allergic and atopic conditions**				
Allergic rhinitis	24 (19.4%)	15 (24.2%)	9 (14.5%)	*p* = 0.173
Asthma	17 (13.7%)	10 (16.1%)	7 (11.3%)	*p* = 0.433
Chronic spontaneous urticarial	11 (8.9%)	3 (4.8%)	8 (12.9%)	*p* = 0.114
Food allergy	14 (11.3%)	6 (9.7%)	8 (12.9%)	*p* = 0.570
Eczema	14 (11.3%)	7 (11.3%)	7 (11.3%)	*p* = 1.000
Drug allergy	49 (40%)	25 (40.3%)	24 (38.7%)	*p* = 0.854
**Vaccine reactions**				
Urticaria only	26 (21%)	14 (22.6%)	12 (19.4%)	*p* = 0.659
Angioedema only	17 (13.7%)	11 (17.7%)	6 (9.7%)	*p* = 0.192
Cutaneous	68 (54.8%)	36 (58.1%)	32 (51.6%)	*p* = 0.470
Upper airway	3 (2.4%)	1 (1.6%)	2 (3.2%)	*p* = 0.559
Lower airway	9 (7.3%)	3 (4.8%)	6 (9.7%)	*p* = 0.299
Cardiovascular	6 (4.8%)	2 (3.2%)	4 (6.5%)	*p* = 0.403
Gastrointestinal	3 (2.4%)	1 (1.6%)	2 (3.2%)	*p* = 0.559
Anaphylaxis	1 (0.8%)	0 (0%)	1 (1.6%)	*p* = 1.000 *
Unknown	2 (1.6%)	0 (0%)	2 (3.2%)	*p* = 0.496 *

Legend: Cutaneous: pruritus, rash (urticarial and non-urticarial), angioedema, flushing; Upper airway: throat swelling, hoarse voice, globus; Lower airway: wheezing, cough, breathlessness; Cardiovascular: tachycardia and hypotension; Gastrointestinal: nausea, vomiting, abdominal pain, diarrhoea; Chi-square or * Fisher exact tests were used for categorical variables; DI: direct inoculation; IDT: intradermal test; SPT: skin prick test.

**Table 2:** Details of the type of vaccine allergies of the 124 patients in the SPT/IDT and DI cohort.

**Polysorbate-Containing Vaccines**	**Number of Patients (%)**
Influenza	54 (43.5%)
Tetanus	31 (25.8%)
Hepatitis B	6 (4.8%)
Pneumococcal	10 (8.1%)
Human Papilloma Virus	6 (4.8%)
Tetanus, Diphtheria and Pertussis	8 (6.5%)
Varicella	1 (0.80%)
Hepatitis A	3 (2.4%)
Meningococcal	1 (0.80%)
**Non-Polysorbate-Containing Vaccines**	**Number of Patients (%)**
Bacillus Calmette–Guérin	3 (2.4%)
Polio	4 (3.2%)
Typhoid	3 (2.4%)
Yellow fever	2 (1.6%)
Rabies	1 (0.80%)
Measles, Mumps, Rubella	7 (5.6%)

**Table 3:** Descriptive statistics of anti-Pfizer BNT162b2 IgE, cytokines and ICAM-1 for endothelial activation pre and post BNT162b2 vaccine.

Laboratory Tests	Reference Ranges	Non-Reactors, *n* = 58		Reactors, *n* = 9		*p*-Value
		Pre-Vaccination	1 h Post Vaccination	Pre-Vaccination	1 h Post Vaccination	
Anti-BNT162b2 IgE (ng/mL)	N.A.	<0.45 (below detection limit)	N.A.	<0.45 (below detection limit)	N.A.	N.D.
IL-4 (pg/mL)	<2	<1 (below detection limit)	<1 (below detection limit)	<1 (below detection limit)	<1 (below detection limit)	N.D.
IL-33 (pg/mL)	<2	1.69 ± 0.95	1.52 ± 0.86	1.74 ± 0.97	1.30 ± 0.26	0.925
C5a (ng/mL)	47.42 ± 31.07	51.96 ± 27.10	50.88 ± 23.14	53.53 ± 30.58	48.18 ± 22.47	0.903
ICAM-1 (ng/mL)	<95	58.80 ± 24.76	55.76 ± 25.83	53.59 ± 17.04	58.35 ± 19.85	0.529

N.A.: not available; N.D.: not done; Mann–Whitney U test; ICAM-1: intercellular adhesion molecule-1; reference data from 21 normal controls; ELISA detection limit, IL-4 and IL-33 (1 pg/mL), C5a and ICAM-1 (0.12 ng/mL), TTSH Immunology Research Laboratory.

## Data Availability

The data presented in this study are available on request from the corresponding author. The data are not publicly available due to privacy and ethical reasons.
